# Correction: A flexible method for optimising sharing of healthcare resources and demand in the context of the COVID-19 pandemic

**DOI:** 10.1371/journal.pone.0251222

**Published:** 2021-04-29

**Authors:** Lucas Lacasa, Robert Challen, Ellen Brooks-Pollock, Leon Danon

The following information is missing from the Funding statement: LD and EBP are supported by Medical Research Council (MRC) (MC/PC/19067). LL acknowledges funding from a QMUL’s COVID Response Grant.
